# Comparison of Hepatic Metabolite Profiles between Infant and Adult Male Mice Using ^1^H-NMR-Based Untargeted Metabolomics

**DOI:** 10.3390/metabo12100910

**Published:** 2022-09-27

**Authors:** Doyoung Kwon, Wonho Lee, Sou Hyun Kim, Young-Suk Jung

**Affiliations:** 1 Department of Pharmacy, Research Institute for Drug Development, College of Pharmacy, Pusan National University, Busan 46241, Korea; 2 Jeju Research Institute of Pharmaceutical Sciences, College of Pharmacy, Jeju National University, Jeju 690-756, Korea

**Keywords:** metabolomics, ^1^H-NMR, liver, age difference, infant, adult, metabolite

## Abstract

Although age-related characteristics of hepatic metabolism are reported, those in infants are not fully understood. In the present study, we performed untargeted metabolomic profiling of the livers of infant (3-week-old) and adult (9-week-old) male ICR mice using ^1^H-NMR spectroscopy and compared 35 abundant hepatic metabolite concentrations between the two groups. The liver/body weight ratio did not differ between the two groups; however, serum glucose, blood urea nitrogen, total cholesterol, and triglyceride concentrations were lower in infants than in adults. Hepatic carbohydrate metabolites (glucose, maltose, and mannose) were higher, whereas amino acids (glutamine, leucine, methionine, phenylalanine, tyrosine, and valine) were lower in infant mice than in adult mice. The concentrations of ascorbate, betaine, sarcosine, and ethanolamine were higher, whereas those of taurine, inosine, and O-phosphocholine were lower in infant mice than in adult mice. The differences in liver metabolites between the two groups could be due to differences in their developmental stages and dietary sources (breast milk for infants and laboratory chow for adults). The above results provide insights into the hepatic metabolism in infants; however, the exact implications of the findings require further investigation.

## 1. Introduction

The liver is an important, multi-functional organ in the human body. Xenobiotics, such as nutrients, drugs, and chemicals, absorbed from the intestine are mainly metabolized in the liver and provided to other tissues and/or excreted from the body [1]. Exogenous toxicants are mostly detoxified in the liver; however, in some cases, they are metabolically activated to generate reactive radicals that cause hepatotoxicity [2,3]. Numerous biomolecules, including proteins and hormones, are synthesized, degraded, and/or transformed in the liver and released into the blood circulation [4,5]. As this organ plays critical roles in the regulation of carbohydrate, amino acid, and lipid metabolism, changes in the metabolic functions of the liver can directly affect the health status of the whole body [6,7,8].

Age-related changes in hepatic drug metabolism in humans have been reported [9,10,11,12], and older populations have low metabolic capacity in their livers [13]. Compared with other age groups, infants show markedly different hepatic metabolism due to developmental immaturity and distinct dietary intake, usually breast milk or baby formula [14,15]. Moreover, the development and progression of liver diseases in the pediatric population differs from those in adults in terms of type and etiology [16].

Metabolite profiling of human biological samples using ^1^H-nuclear magnetic resonance (NMR) and/or mass spectrometry (MS) has been used to provide snapshots of human metabolism [17]. Serum and/or urine samples from children show different metabolite profiles than those from adults [18]. Intriguingly, even in early childhood, significant changes in biological metabolites have been found between infants and children owing to their rapid growth [19,20,21,22]. However, because of the relatively small sample sizes of pediatric patients, the metabolic phenotypes of these young age groups have been less characterized than those of adults [22].

Compared to non-invasive biological samples such as blood and urine, the use of human liver tissues has been extremely limited in metabolomic research owing to its lower availability. Instead, animal livers have been used to analyze hepatic metabolomes, and their age-dependent characteristics have been identified in several studies [23,24,25]. As aging is considered the primary risk factor for the development of human diseases, metabolite profiling of the liver of elderly animals has been performed in most studies [23,26]. However, liver metabolomics of infantile/pediatric animals has scarcely been investigated.

Recently, we reported that infant mice are much more susceptible to acetaminophen-induced hepatotoxicity than adult mice because of higher concentrations of phase I drug-metabolizing enzymes and lower concentrations of antioxidant enzymes in their livers [27]. However, differences in the hepatic metabolome between the two age groups remain unclear. Therefore, in the present study, we compared the liver metabolite profiles of infant and adult mice. Liver tissues collected from 3- and 9-week-old male mice were analyzed by untargeted metabolomics using ^1^H-NMR spectroscopy, and the abundant small molecules were quantified. The results showed significant differences in the hepatic metabolite concentrations between the two groups, including carbohydrates, amino acids, and their derivatives.

## 2. Materials and Methods

### 2.1. Animals

Male ICR mice of different ages (2- and 8-week-old) were purchased from Hyochang Science (Daegu, Korea). Female mice were not used because the hormonal fluctuation can affect the results. Animal experiments were performed according to protocols approved by the Animal Care Use Committee of Pusan National University (PNU-2018-1860). The animal facility was maintained at a controlled temperature (22 ± 2 °C), humidity (55 ± 5 °C), and 12-h dark/light cycle. The mice were acclimated for one week, and the infant mice were housed with their mother female mice during this period. Rodent chow (Sam #31, Samtako Bio Korea, Osan, Korea) was provided, and its nutritional composition is shown in Table 1. Mice aged 3 (infant, n = 9) and 9 (adult, n = 9) weeks were sacrificed, and the blood and liver were collected. Liver tissues were weighed and stored at −80 °C.

### 2.2. Serum Biochemical Analysis

Serum was separated from whole blood by centrifugation using BD Microtainer Serum Collection Tubes (Becton Dickinson and Company, Franklin Lakes, NJ, USA). The serum activities of alanine transaminase (ALT) and aspartate transaminase (AST) and the concentrations of albumin (ALB), total protein (TP), creatinine (Crea), blood urea nitrogen (BUN), total cholesterol (TC), triglycerides (TG), glucose (GLU), low-density lipoproteins, and high-density lipoproteins were determined using an automated chemistry analyzer (Prestige 24i, Tokyo Boeki Medisys Inc., Tokyo, Japan).

### 2.3. ^1^H HR-MAS NMR Measurements

Intact liver tissues were analyzed using ^1^H high-resolution magic-angle spinning nuclear magnetic resonance (HR-MAS NMR). Liver tissue (25 mg) was transferred into a 4 mm HR-MAS rotor, and D_2_O (25 μL) containing 2 mM sodium 3-trimethylsilyl-2,2,3,3-d4-propionate (TSP-d4) was added. Spectral data were acquired using an Agilent 600 MHz NMR spectrometer (Agilent Technologies, Santa Clara, CA, USA) equipped with a gHX NANO probe using Vnmrj 4.2 software. The rotor was spun at a magic angle (θ = 54.74°) at 2060 Hz. The temperature was set at 297 K. A Carr–Purcell–Meiboom–Gill (CPMG) pulse sequence with water peak pre-saturation was used to suppress the signals of macromolecules, lipoproteins, and water. Each spectrum was acquired using 128 scans with a spectral width of 9615.4 Hz, an acquisition time of 3.0 s, and a relaxation delay of 3.0 s.

### 2.4. Spectral Pre-Processing

The obtained ^1^H-NMR spectra were phased, baseline-corrected, calibrated, and quantified using Chenomx NMR Suite 7.1 software (Chenomx Inc., Edmonton, AB, Canada) that includes a pH-dependent 600 MHz library database, which is an integrated set of tools for processing and quantifying NMR spectra. TSP-d4 was used as the chemical shift and quantitation standard. For accurate metabolite identification, single and overlapping signals were confirmed using spike-in experiments and 2D correlation spectroscopy (COSY).

### 2.5. Statistical Analysis

All results are expressed as the mean ± SE and were analyzed using a two-tailed unpaired Student’s *t*-test. An acceptable level of significance was established at a *p* value < 0.05. The metabolomic data were normalized using probabilistic quotient normalization (PQN), and statistical and pathway analyses were performed on the quantified metabolite data using MetaboAnalyst 4.0 software (http://www.metaboanalyst.ca; Edmonton, AB, Canada; accessed on 6 October 2020). The normalized values were scaled using the square root of the standard deviation (Pareto scaling) prior to multivariate statistical analyses, including partial least squares discriminant analysis (PLS-DA), heatmap hierarchical clustering, and pathway analyses. The PLS-DA results were visualized as scatter scores and variable importance in projection (VIP) plots. Heatmap hierarchical clustering, which shows the metabolic similarity between individual liver samples, was analyzed using Pearson distance and the Ward clustering algorithm. Biochemical pathway analysis was conducted using algorithms that consisted of a global test and relative-betweenness centrality for the analyses of pathway enrichment and topology, respectively.

## 3. Results

### 3.1. Body and Liver Weights of Infant and Adult Mice

**Figure 1:**
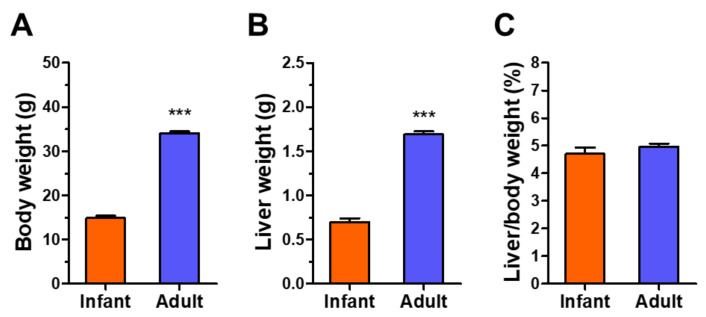
Body and liver weights and the ratio of liver/body weight of infant (3-week-old, n = 9) and adult (9-week-old, n = 9) male mice: (**A**) Body weight; (**B**) Liver weight; (**C**) Liver/body weight ratio. Data are presented as mean ± SE. Student’s *t*-test, *** *p* < 0.001, vs. infant.

Before analyzing hepatic metabolites, we observed general differences between infant and adult mice. The body and liver weights of adult mice were more than two-fold higher than those of infant mice (Figure 1A,B); however, the relative liver weights were not different between the two groups (Figure 1C).

### 3.2. Serum Biomarkers

**Figure 2:**
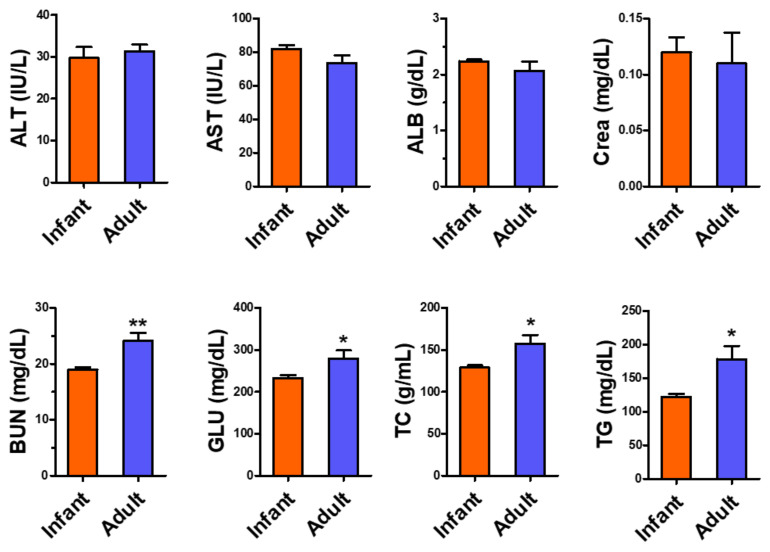
Serum biomarkers of infant (3-week-old, n = 9) and adult (9-week-old, n = 9) male mice. Data are presented as mean ± SE. Student’s *t*-test, *,** *p* < 0.05, and 0.01, respectively, vs infant. ALT, alanine transaminase; AST, aspartate transaminase; ALB, albumin; Crea, creatinine; BUN, blood urea nitrogen; GLU, glucose; TC, total cholesterol; TG, triglyceride.

Serum biomarkers of liver injury and ALT and ALT activities were not different between infant and adult mice (Figure 2). The ALB and Crea concentrations did not show any differences, but the BUN concentration, indicating kidney function, was higher in adult mice than in infant mice. Blood GLU, TC, and TG concentrations were lower in infant mice than in adult mice.

### 3.3. Hepatic Metabolite Profiling Using ^1^H-NMR

**Figure 3:**
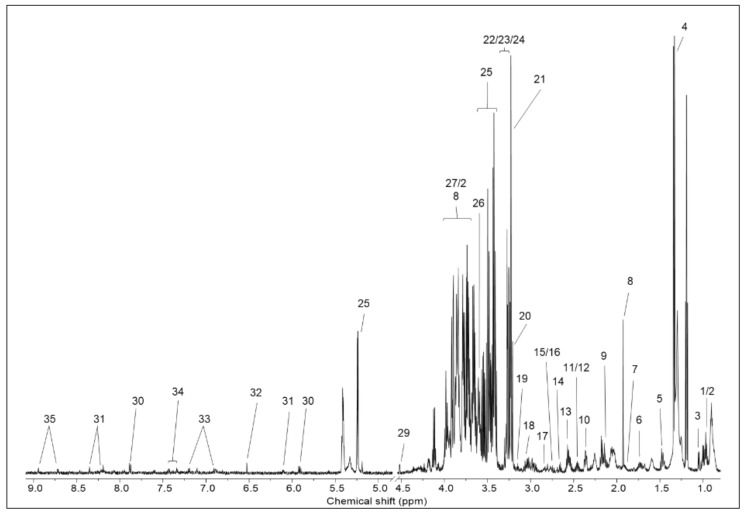
Representative 600 MHz 1H NMR spectra of the liver. Key: 1, isoleucine; 2, leucine; 3, valine; 4, lactate; 5, alanine; 6, Lysine; 7, ornithine; 8, acetate; 9, methionine; 10, glutamate; 11, glutamine; 12, carnitine; 13, glutathione; 14, ß-alanine; 15, dimethylamine; 16, sarcosine; 17, carnosine; 18, creatine; 19, ethanolamine; 20, choline; 21, O-phosphocholine; 22, sn-glycerol-3-phosphocholine; 23, taurine; 24, betaine; 25, glucose; 26, glycine; 27, maltose; 28, mannose; 29, ascorbate; 30; uridine; 31, inosine; 32, fumarate; 33, tyrosine; 34, phenylalanine; 35, niacinamide.

**Figure 4:**
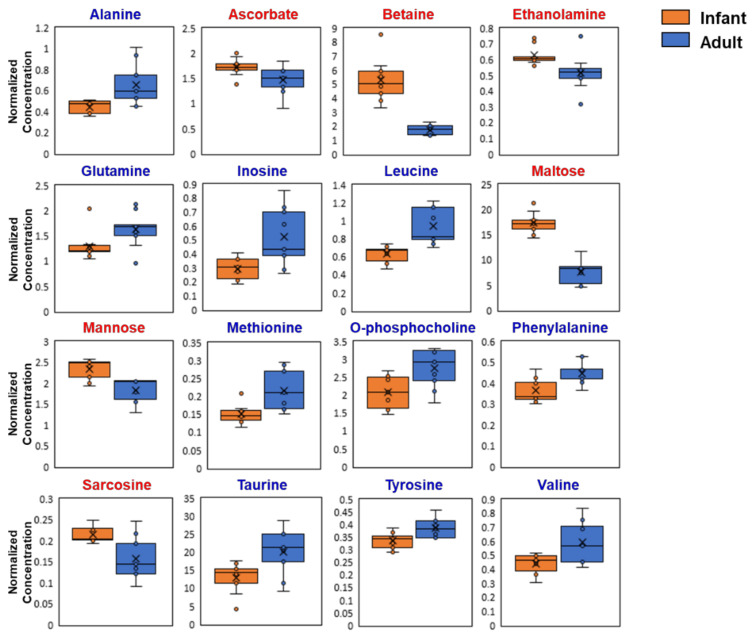
Boxplot of the statistically important hepatic metabolites (*p* < 0.05) in infant (3-week-old, n = 9) and adult (9-week-old, n = 9) male mice. Metabolites written in red letters are higher in the infants, while those in blue letters are higher in the adults.

The metabolite profiles of infant and adult mouse liver samples were analyzed using ^1^H-NMR. Representative ^1^H-NMR spectra of an adult mouse liver with the assigned metabolites are shown in Figure 3. A total of 35 detected metabolites were quantified, and their relative concentrations are listed in Table 2. Statistical analysis using Student’s *t*-test showed that the hepatic concentrations of 16 metabolites were significantly different between the infant and adult mice groups (Table 2 and Figure 4). Infant mice had higher concentrations of ascorbate, betaine, ethanolamine, maltose, mannose, and sarcosine in their livers than adult mice did. In contrast, the concentrations of several amino acids (alanine, glutamine, leucine, methionine, phenylalanine, tyrosine, and valine), inosine, O-phosphocholine, and taurine were higher in adult mice than in infant mice (Figure 4).

### 3.4. Multivariate Analysis

**Figure 5:**
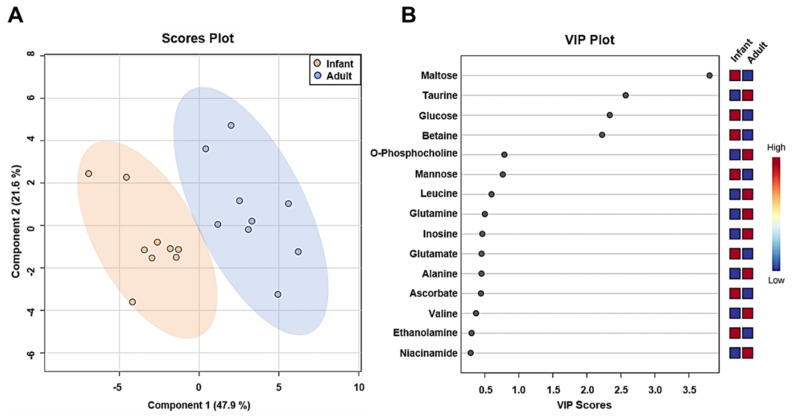
PLS−DA and VIP score plots. (**A**) PLS−DA score plot comparing infant (3−week−old, n = 9) and adult (9−week−old, n = 9) male mice groups. (**B**) The variable importance in projection (VIP) score plot from PLS−DA analysis of important metabolites from infant and adult groups.

**Figure 6:**
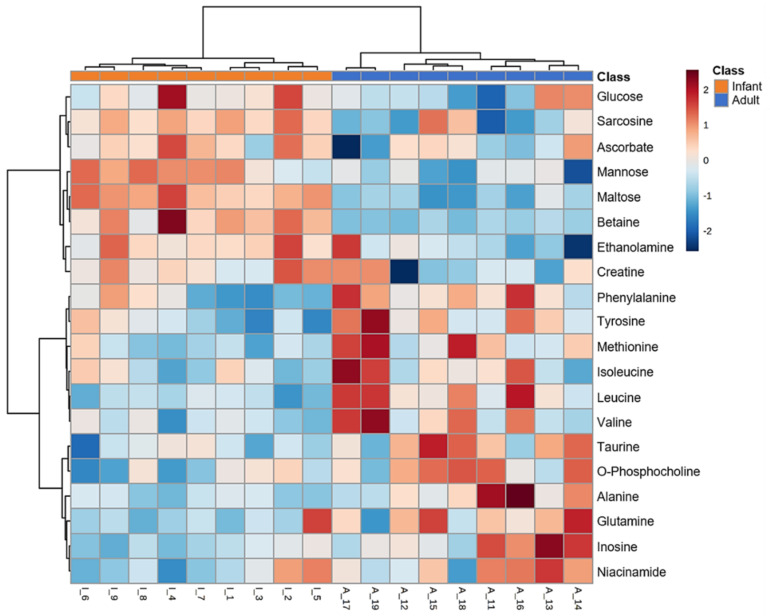
Heatmap hierarchical clustering analysis for top 20 metabolites that differed among infant (3−week−old, n = 9) and adult (9−week−old, n = 9) male mice. Red and blue colors indicate fold higher and lower relative concentrations of metabolites, respectively.

The results of PLS-DA are shown as a score scatter (Figure 5A) and VIP plot (Figure 5B) to differentiate the patterns of metabolites between the groups and identify the metabolites that mainly contribute to differentiation. To avoid the risk of overfitting, 10-fold cross-validation was used in this model. In the PLS-DA score plot (R2 = 0.938, Q2 = 0.756), the first principal component (47.9 %) distinctly separated infant and adult groups (Figure 5A). The VIP scores indicated the top 15 metabolites contributing to the differentiation of the two groups in the PLS-DA model (Figure 5B). Colored boxes indicate higher (red) or lower (blue) relative concentrations of the corresponding metabolites in each group (Figure 5B). Maltose, taurine, GLU, and betaine, which had VIP scores higher than 1, were considered the most significant metabolites for differentiating between the two groups. Heatmap hierarchical clustering based on the hepatic metabolites showed separated patterns between infant and adult mice, indicating the strong similarity of the metabolic profile in each group (Figure 6). These results from the PLS-DA and heatmap hierarchical clustering analysis demonstrated that infants have significantly different metabolite phenotypes in their livers than adults.

### 3.5. Metabolic Pathway Analysis

**Figure 7:**
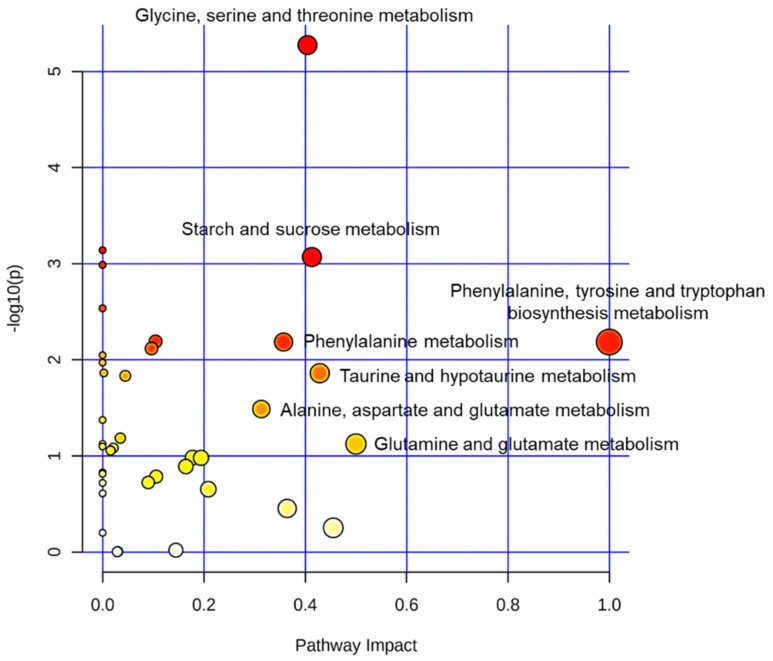
Metabolite sets enrichment analysis. The impacts and significances of biochemical pathways affected by different metabolite profiles of infant (3−week−old, n = 9) and adult (9−week−old, n = 9) male mice livers. The color and size of each node indicate the *p* value and pathway impact value, respectively. Darker red color indicates higher *p* value.

A total of 41 biochemical pathways involving the detected metabolites are plotted in Figure 7 according to their impact (x-axis) and statistical significance (y-axis). Among them, age-dependent differences (*p* < 0.05) and pathway impacts >0.2 were found in phenylalanine, tyrosine, and tryptophan biosynthesis metabolism; glutamine and glutamate metabolism; taurine and hypotaurine metabolism; starch and sucrose metabolism; glycine, serine, and threonine metabolism; phenylalanine metabolism; and alanine, aspartate, and glutamate metabolism (Figure 7).

## 4. Discussion

Aging results in a decline in biological functions due to the decay of biomolecules in the late period of life [17]. Growth is a synthetic process of macromolecules from simple nutrients using energy to mature organismal functions in the early stages of life [17,28,29]. These distinguishable phenomena, growth and aging, are closely linked, and health status during childhood can influence the development of diseases in adulthood [28,29]. Neonates and infants undergo the most rapid development after birth; thus, understanding the dynamic changes of cellular processes in early childhood is important for maintaining human health. [20]. Metabolomic analyses can present a snapshot of biological metabolism affected by genetic, transcriptomic, proteomic, and environmental factors [17]. Age is also an important factor influencing human metabolism; however, growth-associated metabolic alterations in pediatrics have been less examined compared to aging-associated changes in the elderly [17]. In the present study, the results obtained using ^1^H-NMR-based untargeted metabolomic analysis using mouse livers showed significant differences in the major hepatic metabolites, including simple sugars, amino acids, and other biomolecules, between infants and adults; however, relative liver weight and liver health status did not change (Figure 1 and Figure 2).

### 4.1. Carbohydrates

Carbohydrates are the main energy source for humans [30]. More energy is needed for human organ development because energy expenditure (kcal/kg body weight) is significantly higher in growing babies and children than in adults [30]. GLU, the major and abundant energy source metabolized to pyruvate for ATP synthesis [31], is an important carbon source for the biosynthesis of fatty acids and amino acids [30]. The liver plays a central role in GLU metabolism [31]. In the fed state, GLU can be stored as hepatic glycogen, which can be broken down into GLU and released into the bloodstream during fasting [31,32]. In humans, energy intake is highest at 107 kcal/kg/day at 6–12 months and gradually decreases to 41 kcal/kg/day in 6–12-year-old children [33]. The size-adjusted energy expenditure of a 1-year-old baby was reportedly the highest in human life, at about 50% above that of adults [34]. In contrast to adults, the primary source of GLU for infants is lactose in breast milk (approximately 7% lactose in milk) [30,35,36]. Human blood GLU concentration is maintained consistently (3.5–5.5 mmol/L) from infanthood to adulthood because this simple sugar is the only energy source for normal brain function [37]. However, the production rate of endogenous GLU is the highest in neonates and gradually decreases with age [30]. The higher GLU synthesis rate during puberty [30] suggests that GLU production is also related to age-dependent energy demand. A progressive decrease in hepatic GLU output, associated with an increase in age due to declining liver function, has also been reported [32]. In our results, the higher hepatic GLU concentration in infants than in adults (*p* = 0.08, Table 2) might be associated with the rapid growth/development-related energy requirement in infant mice. Intriguingly, blood GLU concentrations were lower in infants than in adults (Figure 1), suggesting different regulation of GLU concentration in the blood and liver.

Mannose is closely associated with GLU metabolism because it can be synthesized from GLU and also can be converted to GLU [38]. Mannose is important for the development of fetuses and neonates [39]. Human breast milk contains approximately 40 mM of free mannose and other oligosaccharides, which contribute to the establishment of gut flora and suppression of pathogen binding to intestinal epithelial cells. [39]. Mannose is used for N- and O-glycosylation; any disorders in these reactions during infancy result in developmental delays [40]. Although mannose concentration (75 µM) in mouse milk is less than that in human breast milk, the plasma concentration of mannose depends on its concentration in the mother’s milk [41]. Thus, the higher mannose concentration in infants (Table 2 and Figure 4) could be related to its intake during lactation.

Maltose, a disaccharide composed of two GLU units, can be broken down into GLU by maltases. Maltose, usually found in germinated grains, can be hydrolyzed from starch by α-amylase in saliva and the small intestine [42]. The reason for the high maltose concentration in the infant mouse liver (Table 2, Figure 5) is unclear, but the higher maltose could be utilized as an energy source in rapidly growing mice. Hepatic GLU and related metabolites, including maltose and maltotetraose, are significantly increased in aged (2-year-old) mice compared to those in young (13-week-old) adults; alteration in hepatic glycogen metabolism is suggested as a biomarker of aging [23]. Decreasing concentrations of GLU, mannose, and maltose with growth (Table 1 and Figure 4) suggest that growth and aging contrarily affect hepatic GLU metabolism.

### 4.2. Amino Acids

Amino acids, the building blocks of proteins, act as signaling molecules in cells [43]. The liver is a major organ involved in amino acid metabolism that regulates circulating amino acid concentrations [43]. Among the 11 detectable amino acids, the concentrations of essential amino acids, including methionine, leucine, valine, and phenylalanine, and non-essential amino acids, including alanine, glutamine, and tyrosine, were lower in infant mice than that in adult mice (Table 2 and Figure 5). Interestingly, no detected amino acids were higher in infants, indicating that free amino acid concentrations in the liver were lower in 3-week-old mice than in adult mice. Hepatic free amino acid concentrations could be affected by the different types of diets for infants and adults (mouse milk and rodent chow), which contain different concentrations of proteins and amino acids. The protein content of rodent chow provided to adult mice was 22.02 % (*w/w*) (Table 1), and the average food consumption of 9-week-old adult mice during the 1-week acclimation period was 0.12 g/day/g body weight (data not shown). Thus, daily protein intake was 26 mg/day/g body weight. The 2-week-old mice were housed with their mothers for 1 week; thus, mouse milk could be the major dietary source for infants. TP concentration in mouse milk was measured as 10–14% by weight [44,45], and the daily milk intake of 15-day-old mice was reported to be 0.15 mL/day/g body weight [46]. Thus, the protein intake of infant mice during the lactation period was estimated to be 15–21 mg/day/g body weight. Adults supplemented more proteins per day relative to their body weights, which may explain the higher amino acid concentrations in the adult livers. Another factor that could influence hepatic amino acid concentrations is the demand for growth. Protein and amino acid requirements (g/kg/day) are higher in infants than in adults due to their rapid growth; amino acid requirements gradually decrease with decreasing growth rate [47,48]. Thus, not only protein/amino acid intake but also utilization of amino acids for protein synthesis can be possible factors resulting in differences in hepatic free amino acid concentrations. A clinical study that measured blood amino acid concentrations in infants (6–12 months old) and children (6–12 years old) showed that the serum concentrations of several amino acids, including glycine, leucine, isoleucine, methionine, and phenylalanine, increased with age [33]. In contrast, gradual decreases in serum concentrations of most amino acids with age were also found in humans aged 32–81 [49]. An animal study also showed reduced plasma alanine, proline, serine, tyrosine, and methionine concentrations in aged (93 weeks) mice compared to young adults (13 weeks) [23]. These results suggest that free amino acid concentrations are important indicators of growth and aging.

### 4.3. Other Metabolites

In addition to simple sugars and amino acids, the hepatic concentrations of several metabolites differed between the infant and adult mice (Table 2 and Figure 5). Ascorbic acid is an essential nutrient known to prevent scurvy in humans [50]. A clinical survey conducted in the US showed that adolescents (12–17 years) have higher plasma ascorbic acid concentrations (46.0 µM and 50.0 µM in males and females, respectively) than adults at the age of 25–44 (36.3 µM and 42.6 µM in males and females, respectively) in both genders, and the concentrations (44.9 µM and 55.1 µM in males and females, respectively) in elderly (65–74 years) increased again [51]. Unlike humans, rodents can synthesize ascorbate mainly in the liver [52]. Age-related changes in hepatic ascorbic acid content in rodents have been reported, and aged mouse livers contain more ascorbic acid than young adult livers [23]. In our study, higher hepatic ascorbate concentrations were observed in infant mice than in adult mice (Table 1 and Figure 5). As ascorbate is important for the development of the brain and maintenance of immune functions [53,54], the higher concentration of ascorbate in infants can be associated with its higher demand for proper growth.

Betaine (trimethyl glycine) acts as an osmolyte and methyl donor in homocysteine and methionine metabolism in the liver [55]. Betaine provides a methyl group to homocysteine by betaine-homocysteine S-methyltransferase (BHMT) to form methionine, which can be further converted to S-adenosylmethionine, a methyl donor, in many methylation reactions [55]. In animals, betaine is synthesized from choline and converted to dimethylglycine, followed by sarcosine, glycine, and serine [55]. Betaine can be obtained from foods, and human breast milk contains betaine at approximately 36.7 µM [56]. Although there is no information on betaine content in mouse milk, higher hepatic betaine concentration in infant mice may be associated with lactation. Because hepatic choline concentrations were not different between the two groups (Table 1), the higher betaine concentration could have originated from milk supplementation; however, the exact relationship remains unclear. Sarcosine, a demethylated betaine product, was also higher in the infant liver, which appears to originate from the betaine. An age-dependent increase in plasma homocysteine concentration was observed in children and adolescents aged 4–19 [57]. Homocysteine, a risk factor for cardiovascular disease, is regulated in the liver and lowered by the reaction with betaine [57]. Thus, the change in betaine concentration in our results might explain the change in homocysteine concentration in childhood, despite the species difference. Betaine is positively related with developmental outcomes [58], suggesting that the higher betaine may be associated with the rapid growth of the infant mice.

Ethanolamine (ETA) is an essential compound usually found in the head of phosphatidylethanolamine (PE) [59]. Mammalian cells cannot synthesize ETA; however, this compound can be obtained from diets such as ETA and/or PE. The free form of ETA is present in the blood at approximately 2 µM; however, human breast milk contains a much higher concentration of ETA (46 µM) [59]. There are no data for ETA content in mouse milk, but ETA intake by lactation could be a reason for the higher ETA concentration in infant mice than in adult mice (Table 1 and Figure 4). ETA accelerates mammalian cell growth via the synthesis of PE and phosphatidylcholine [59]; thus, a higher hepatic ETA concentration seems to be needed to stimulate the growth of infant mice.

In contrast to ascorbate and ETA, several molecules, such as taurine, inosine, and O-phosphocholine, were lower in infant mice than in adult mice (Table 2 and Figure 5). Taurine is a cellular osmolyte that acts as a substrate for bile acids. We previously reported that infant mice show lower hepatic taurine concentration than adult mice due to the lower protein level of cysteine dioxygenase (CDO), the rate-limiting enzyme for taurine synthesis [27]. The lower taurine concentration was thought to be related to the utilization of cysteine for glutathione (GSH) synthesis [27]. Inosine, a metabolite of purine metabolism generated from adenosine or inosine monophosphate, was higher in adult livers than in infant livers (Table 2 and Figure 4). Hepatic inosine concentration was higher in old mice than in adult mice [23], suggesting that purine metabolism is consistently altered to increase inosine concentration during both growth and aging periods. O-Phosphocholine, an intermediate of choline metabolism, was low in infants, although choline concentrations were not different between the two groups. The exact reasons for these changes are uncertain; however, the distinguishable metabolite profiles between the two groups (Figure 5, Figure 6 and Figure 7) suggest that infant and adult mice have significantly different metabolic processes in their livers.

## 5. Conclusions

In the present study, we determined the major metabolites in the liver of infant and adult male mice by untargeted metabolomic analysis using ^1^H-NMR spectroscopy. The results showed that the hepatic metabolite concentrations were significantly different between infants and adults. Simple sugars, ascorbic acid, betaine, sarcosine, and ethanolamine concentrations were higher, but amino acid and taurine concentrations were lower in infant mice than in adult mice. The exact reasons for the difference in each metabolite concentration between the groups are unclear; however, the differences in diet (mouse milk or rodent chow), growth rate, and maturity can be possible factors affecting their concentrations. The present findings from the liver-specific analysis may provide useful information for understanding the hepatic metabolism of pediatric patients. Previous age-related animal studies have mostly focused on aging; however, our study is the first to show growth-related changes in the hepatic metabolome. Nevertheless, absolute quantitation of the major metabolites by targeted metabolomics and investigation of the female liver metabolome are needed to clarify the exact relationship between age and hepatic metabolism in the early stages of life.

## Figures and Tables

**Table 1:** Nutritional composition of rodent chow.

Nutrients	Weight (%)	Calories (%)
Protein	22.02	21.6
Carbohydrates	65.96	64.85
Fat	6.10	13.49
Calcium	0.81	-
Phosphorus	0.70	-
Ash	4.41	-
Total	100	100

**Table 2:** Relative concentrations of metabolites acquired using ^1^H-NMR spectroscopy from infant (3-week-old, n = 9) and adult (9-week-old, n = 9) male mice livers.

	Infant			Adult			Percent Change(%)	*p*-Value	
	Mean	±	SE	Mean	±	SE			
Acetate	2.203	±	0.066	2.201	±	0.163	−0.10	0.991	
Alanine	0.444	±	0.021	0.656	±	0.068	47.66	0.009	**
Ascorbate	1.721	±	0.061	1.469	±	0.097	−14.67	0.042	*
Betaine	5.317	±	0.520	1.742	±	0.125	−67.24	0.000	***
Carnitine	0.702	±	0.036	0.687	±	0.026	−2.20	0.735	
Carnosine	0.610	±	0.023	0.561	±	0.038	−7.95	0.290	
Choline	0.926	±	0.051	0.985	±	0.072	6.30	0.519	
Creatine	0.228	±	0.006	0.203	±	0.011	−10.83	0.077	
Dimethylamine	0.086	±	0.005	0.081	±	0.007	−5.17	0.602	
Ethanolamine	0.628	±	0.020	0.519	±	0.039	−17.38	0.023	*
Fumarate	0.360	±	0.019	0.361	±	0.035	0.18	0.987	
Glucose	50.511	±	2.770	42.517	±	3.250	−15.83	0.080	
Glutamate	3.341	±	0.216	2.948	±	0.190	−11.77	0.191	
Glutamine	1.301	±	0.097	1.629	±	0.117	25.19	0.047	*
Glutathione	1.592	±	0.068	1.713	±	0.151	7.57	0.477	
Glycine	2.305	±	0.072	2.185	±	0.125	−5.20	0.420	
Inosine	0.297	±	0.027	0.521	±	0.071	75.58	0.009	**
Isoleucine	0.329	±	0.014	0.382	±	0.025	15.90	0.088	
Lactate	11.171	±	0.694	10.909	±	0.726	−2.35	0.797	
Leucine	0.631	±	0.031	0.940	±	0.068	49.03	0.001	***
Lysine	0.963	±	0.047	0.870	±	0.039	−9.66	0.149	
Maltose	17.348	±	0.718	7.568	±	0.778	−56.37	0.000	***
Mannose	2.347	±	0.084	1.835	±	0.096	−21.84	0.001	**
Methionine	0.152	±	0.009	0.217	±	0.019	42.84	0.006	**
Niacinamide	0.939	±	0.053	1.074	±	0.058	14.33	0.105	
O-Phosphocholine	2.086	±	0.155	2.764	±	0.190	32.49	0.014	*
Ornithine	0.591	±	0.026	0.593	±	0.073	0.36	0.979	
Phenylalanine	0.368	±	0.020	0.446	±	0.018	21.33	0.010	**
Sarcosine	0.216	±	0.006	0.158	±	0.017	−26.79	0.005	**
Taurine	12.938	±	1.435	20.146	±	2.175	55.71	0.014	*
Tyrosine	0.335	±	0.011	0.385	±	0.013	14.85	0.011	*
Uridine	0.561	±	0.017	0.565	±	0.029	0.62	0.919	
Valine	0.441	±	0.024	0.595	±	0.052	34.87	0.017	*
sn-Glycero−3-phosphocholine	1.007	±	0.056	0.881	±	0.063	−12.53	0.153	
β-Alanine	0.461	±	0.064	0.418	±	0.059	−9.30	0.629	

Mean ± SE. Student’s *t*-test; *, **, *** *p* < 0.05, 0.01, and 0.001, respectively, vs. infants.

## Data Availability

The data presented in this study are available in article.
